# Bis(3-amino­phen­yl) sulfone acetonitrile solvate

**DOI:** 10.1107/S1600536808014840

**Published:** 2008-05-21

**Authors:** Wei Yao, Fang-Shi Li, Da-Sheng Yu, Chui Lu, Jin-Na Zhu

**Affiliations:** a Department of Applied Chemistry, College of Science, Nanjing University of Technolgy, Xinmofan Road No. 5 Nanjing, Nanjing 210009, People’s Republic of China

## Abstract

In the sulfone mol­ecule of the title compound, C_12_H_12_N_2_O_2_S·C_2_H_3_N, the two benzene rings are oriented at a dihedral angle of 80.69 (3)°. Weak intra­molecular C—H⋯O hydrogen bonds result in the formation of two five-membered rings, which both have envelope conformations. In the crystal structure, inter­molecular N—H⋯O hydrogen bonds link the mol­ecules.

## Related literature

For related literature, see: Yang *et al.* (2003 ▶); Rudyk *et al.* (2003 ▶); Ayyangar *et al.* (1981 ▶). For bond-length data, see: Allen *et al.* (1987 ▶).
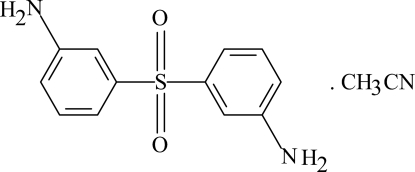


## Experimental

### 

#### Crystal data

C_12_H_12_N_2_O_2_S·C_2_H_3_N
*M* *_r_* = 289.35
Orthorhombic, 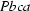

*a* = 9.1690 (18) Å
*b* = 15.559 (3) Å
*c* = 20.960 (4) Å
*V* = 2990.2 (10) Å^3^
*Z* = 8
Mo *K*α radiation
μ = 0.22 mm^−1^
*T* = 294 (2) K
0.40 × 0.30 × 0.30 mm

#### Data collection

Enraf–Nonius CAD-4 diffractometer
Absorption correction: ψ scan (North *et al.*, 1968 ▶) *T*_min_ = 0.917, *T*_max_ = 0.937
2674 measured reflections
2674 independent reflections
1644 reflections with *I* > 2σ(*I*)
3 standard reflections frequency: 120 min intensity decay: none

#### Refinement

*R*[*F* ^2^ > 2σ(*F* ^2^)] = 0.055
*wR*(*F* ^2^) = 0.135
*S* = 1.03
2674 reflections
175 parameters
H-atom parameters constrained
Δρ_max_ = 0.36 e Å^−3^
Δρ_min_ = −0.23 e Å^−3^

### 

Data collection: *CAD-4 Software* (Enraf–Nonius, 1989 ▶); cell refinement: *CAD-4 Software*; data reduction: *XCAD4* (Harms & Wocadlo, 1995 ▶); program(s) used to solve structure: *SHELXS97* (Sheldrick, 2008 ▶); program(s) used to refine structure: *SHELXL97* (Sheldrick, 2008 ▶); molecular graphics: *SHELXTL* (Sheldrick, 2008 ▶) and *PLATON* (Spek, 2003 ▶); software used to prepare material for publication: *SHELXTL*.

## Supplementary Material

Crystal structure: contains datablocks I, global. DOI: 10.1107/S1600536808014840/hk2464sup1.cif

Structure factors: contains datablocks I. DOI: 10.1107/S1600536808014840/hk2464Isup2.hkl

Additional supplementary materials:  crystallographic information; 3D view; checkCIF report

## Figures and Tables

**Table 1:** Hydrogen-bond geometry (Å, °)

*D*—H⋯*A*	*D*—H	H⋯*A*	*D*⋯*A*	*D*—H⋯*A*
C6—H6*A*⋯O1	0.93	2.53	2.913 (4)	105
C8—H8*A*⋯O2	0.93	2.53	2.906 (4)	104
N1—H1*B*⋯O1^i^	0.86	2.32	3.147 (5)	161
N2—H2*B*⋯O2^ii^	0.86	2.28	3.079 (4)	155

Symmetry codes: (i) 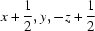; (ii) 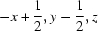.

**Fig. 1.**
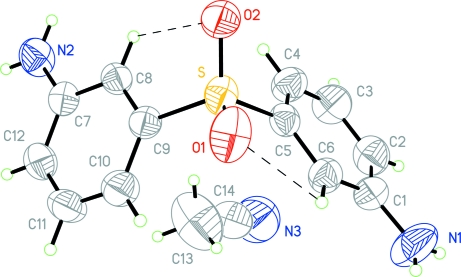
The molecular structure of the title molecule, with the atom-numbering scheme. Displacement ellipsoids are drawn at the 50% probability level. Hydrogen bonds are shown as dashed lines.

**Fig. 2.**
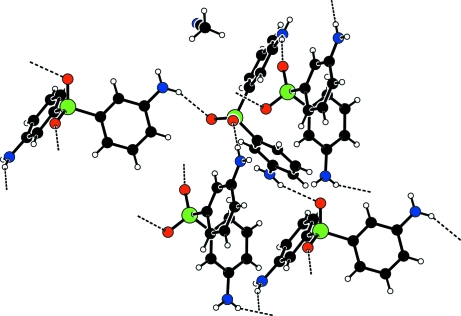
A partial packing diagram of (I). Hydrogen bonds are shown as dashed lines.

## supplementary crystallographic information

### Comment

The title compound, (I), is used for preparing diimide-dicarboxylic acid (Yang *et al.*,  2003) and corresponding truncated dyes analogs of Congo red (Rudyk *et al.*,  2003). As part of our studies in this area, we report herein the synthesis and crystal structure of the title compound, (I).

In the molecule of (I) (Fig. 1), the bond lengths (Allen *et al.*, 1987) and angles are within normal ranges. The asymmetric unit also contains one acetonitrile solvent molecule. Rings A (C1-C6) and B (C7-C12) are, of course, planar and they are oriented at a dihedral angle of A/B = 80.69 (3)°. The weak intramolecular C-H···O hydrogen bonds (Table 1) result in the formation of two five-membered rings C (S/C5/C6/H6A) and D (S/C8/C9/H8A). They adopt envelope conformations, with O1 and O2 atoms displaced by 0.251 (3) and -0.529 (3) Å from the planes of the other ring atoms, respectively.

In the crystal structure, intermolecular N1-H1B···O1^i^ [H1B···O1 2.32 Å, N1···O1 3.147 (3) Å and N1-H1B···O1 161.0°] and N2-H2B···O2^ii^ [H2B···O2 2.28 Å, N2···O2 3.079 (3) Å and N2-H2B···O2 155.0°] hydrogen bonds [symmetry codes: (i) x + 1/2, y, 1/2 - z; (ii) 1/2 - x, y - 1/2, z] link the molecules (Fig. 2), in which they may be effective in the stabilization of the structure.

### Experimental

The title compound, (I), was prepared according to the literature method (Ayyangar *et al.*,  1981). Crystals suitable for X-ray analysis were obtained by dissolving (I) (0.2 g) in acetonitrile (25 ml) and evaporating the solvent slowly at room temperature for about 7 d.

### Refinement

H atoms were positioned geometrically, with N-H = 0.86 Å (for NH_2_) and C-H = 0.93 and 0.96 Å for aromatic and methyl H, respectively, and constrained to ride on their parent atoms with U_iso_(H) = xU_eq_(C,N), where x = 1.5 for methyl H, and x = 1.2 for all other H atoms.

### Crystal data

**Table:**

C_12_H_12_N_2_O_2_S·C_2_H_3_N	*F*_000_ = 1216
*M**_r_* = 289.35	*D*_x_ = 1.285 Mg m^−^^3^
Orthorhombic, *P**b**c**a*	Mo *K*α radiation λ = 0.71073 Å
Hall symbol: -P 2ac 2ab	Cell parameters from 25 reflections
*a* = 9.1690 (18) Å	θ = 10–13º
*b* = 15.559 (3) Å	µ = 0.22 mm^−^^1^
*c* = 20.960 (4) Å	*T* = 294 (2) K
*V* = 2990.2 (10) Å^3^	Block, light yellow
*Z* = 8	0.40 × 0.30 × 0.30 mm

### Data collection

**Table:**

Enraf–Nonius CAD-4 diffractometer	*R*_int_ = 0.0000
Radiation source: fine-focus sealed tube	θ_max_ = 25.1º
Monochromator: graphite	θ_min_ = 1.9º
*T* = 294(2) K	*h* = 0→10
ω/2θ scans	*k* = 0→18
Absorption correction: ψ scan(North *et al.*, 1968)	*l* = 0→24
*T*_min_ = 0.917, *T*_max_ = 0.937	3 standard reflections
2674 measured reflections	every 120 min
2674 independent reflections	intensity decay: none
1644 reflections with *I* > 2σ(*I*)	

### Refinement

**Table:**

Refinement on *F*^2^	Secondary atom site location: difference Fourier map
Least-squares matrix: full	Hydrogen site location: inferred from neighbouring sites
*R*[*F*^2^ > 2σ(*F*^2^)] = 0.055	H-atom parameters constrained
*wR*(*F*^2^) = 0.135	*w* = 1/[σ^2^(*F*_o_^2^) + (0.05*P*)^2^ + 2*P*] where *P* = (*F*_o_^2^ + 2*F*_c_^2^)/3
*S* = 1.03	(Δ/σ)_max_ < 0.001
2674 reflections	Δρ_max_ = 0.36 e Å^−^^3^
175 parameters	Δρ_min_ = −0.23 e Å^−^^3^
Primary atom site location: structure-invariant direct methods	Extinction correction: none

### Special details

**Table:**

Geometry. All e.s.d.'s (except the e.s.d. in the dihedral angle between two l.s. planes) are estimated using the full covariance matrix. The cell e.s.d.'s are taken into account individually in the estimation of e.s.d.'s in distances, angles and torsion angles; correlations between e.s.d.'s in cell parameters are only used when they are defined by crystal symmetry. An approximate (isotropic) treatment of cell e.s.d.'s is used for estimating e.s.d.'s involving l.s. planes.
Refinement. Refinement of F^2^ against ALL reflections. The weighted R-factor wR and goodness of fit S are based on F^2^, conventional R-factors R are based on F, with F set to zero for negative F^2^. The threshold expression of F^2^ > 2sigma(F^2^) is used only for calculating R-factors(gt) etc. and is not relevant to the choice of reflections for refinement. R-factors based on F^2^ are statistically about twice as large as those based on F, and R- factors based on ALL data will be even larger.

### Fractional atomic coordinates and isotropic or equivalent isotropic displacement parameters (Å^2^)

**Table:**

	*x*	*y*	*z*	*U*_iso_*/*U*_eq_	
S	0.45811 (9)	0.20077 (5)	0.17605 (4)	0.0493 (3)	
O1	0.5131 (3)	0.23557 (15)	0.23465 (12)	0.0692 (8)	
O2	0.3393 (3)	0.24395 (14)	0.14448 (13)	0.0662 (7)	
N1	0.9991 (3)	0.2160 (3)	0.11603 (18)	0.0941 (13)	
H1B	1.0185	0.2305	0.1547	0.113*	
H1C	1.0683	0.2116	0.0885	0.113*	
N2	0.1514 (3)	−0.06128 (19)	0.11889 (14)	0.0639 (9)	
H2B	0.1248	−0.1138	0.1240	0.077*	
H2C	0.1096	−0.0298	0.0905	0.077*	
N3	0.8308 (6)	−0.0861 (3)	0.0059 (2)	0.125	
C1	0.8592 (4)	0.2000 (2)	0.09841 (18)	0.0560 (9)	
C2	0.8255 (5)	0.1794 (2)	0.03521 (19)	0.0657 (11)	
H2D	0.9005	0.1746	0.0056	0.079*	
C3	0.6825 (5)	0.1660 (3)	0.01564 (19)	0.0690 (11)	
H3A	0.6633	0.1513	−0.0266	0.083*	
C4	0.5695 (4)	0.1741 (2)	0.05805 (17)	0.0582 (10)	
H4A	0.4734	0.1665	0.0450	0.070*	
C5	0.6022 (3)	0.19405 (19)	0.12110 (15)	0.0428 (8)	
C6	0.7437 (4)	0.2064 (2)	0.14153 (16)	0.0502 (8)	
H6A	0.7622	0.2189	0.1841	0.060*	
C7	0.2607 (4)	−0.0275 (2)	0.15608 (15)	0.0454 (8)	
C8	0.3005 (3)	0.05871 (19)	0.14943 (15)	0.0434 (8)	
H8A	0.2563	0.0927	0.1185	0.052*	
C9	0.4056 (3)	0.09322 (19)	0.18883 (15)	0.0431 (8)	
C10	0.4737 (4)	0.0444 (2)	0.23542 (17)	0.0582 (10)	
H10A	0.5433	0.0685	0.2623	0.070*	
C11	0.4356 (4)	−0.0407 (2)	0.24077 (19)	0.0650 (11)	
H11A	0.4818	−0.0749	0.2711	0.078*	
C12	0.3309 (4)	−0.0763 (2)	0.20243 (17)	0.0563 (9)	
H12A	0.3064	−0.1339	0.2074	0.068*	
C13	0.6947 (7)	−0.0534 (4)	0.1078 (2)	0.130 (2)	
H13A	0.6632	−0.1062	0.1270	0.195*	
H13B	0.6113	−0.0182	0.0986	0.195*	
H13C	0.7580	−0.0234	0.1368	0.195*	
C14	0.7706 (5)	−0.0714 (3)	0.0506 (2)	0.0858 (14)	

### Atomic displacement parameters (Å^2^)

**Table:**

	*U*^11^	*U*^22^	*U*^33^	*U*^12^	*U*^13^	*U*^23^
S	0.0474 (5)	0.0365 (4)	0.0641 (6)	−0.0012 (4)	0.0097 (5)	−0.0078 (4)
O1	0.0785 (18)	0.0611 (15)	0.0680 (17)	−0.0187 (14)	0.0159 (15)	−0.0280 (13)
O2	0.0520 (14)	0.0409 (13)	0.106 (2)	0.0105 (12)	0.0076 (15)	0.0041 (13)
N1	0.0447 (19)	0.153 (4)	0.085 (3)	−0.007 (2)	0.0034 (18)	0.003 (2)
N2	0.075 (2)	0.0524 (17)	0.064 (2)	−0.0164 (16)	−0.0116 (18)	0.0000 (15)
N3	0.125	0.125	0.125	0.000	0.000	0.000
C1	0.045 (2)	0.060 (2)	0.063 (2)	0.0023 (19)	0.0006 (18)	0.0063 (19)
C2	0.064 (3)	0.073 (3)	0.060 (3)	0.006 (2)	0.019 (2)	0.003 (2)
C3	0.072 (3)	0.088 (3)	0.046 (2)	−0.002 (2)	0.002 (2)	−0.004 (2)
C4	0.051 (2)	0.069 (2)	0.054 (2)	−0.0041 (19)	−0.0050 (19)	−0.0003 (18)
C5	0.0463 (18)	0.0338 (16)	0.048 (2)	−0.0005 (15)	0.0029 (16)	0.0007 (15)
C6	0.0490 (19)	0.0523 (19)	0.049 (2)	−0.0045 (18)	−0.0020 (18)	−0.0006 (17)
C7	0.0461 (19)	0.0424 (18)	0.048 (2)	−0.0019 (16)	0.0052 (17)	−0.0044 (15)
C8	0.0448 (19)	0.0408 (17)	0.0447 (18)	0.0043 (15)	0.0052 (16)	0.0023 (15)
C9	0.0413 (18)	0.0376 (17)	0.050 (2)	0.0034 (14)	0.0030 (16)	−0.0015 (15)
C10	0.059 (2)	0.053 (2)	0.063 (2)	−0.0074 (18)	−0.013 (2)	0.0013 (18)
C11	0.070 (3)	0.055 (2)	0.069 (2)	−0.005 (2)	−0.021 (2)	0.0174 (19)
C12	0.064 (2)	0.0436 (19)	0.061 (2)	−0.0034 (19)	0.002 (2)	0.0042 (17)
C13	0.182 (7)	0.111 (4)	0.097 (4)	0.030 (4)	0.051 (4)	−0.002 (3)
C14	0.092 (4)	0.087 (3)	0.078 (3)	0.013 (3)	−0.002 (3)	0.004 (3)

### Geometric parameters (Å, °)

**Table:**

S—O1	1.434 (2)	C5—C6	1.380 (4)
S—O2	1.441 (2)	C6—H6A	0.9300
S—C5	1.756 (3)	C7—C12	1.390 (5)
S—C9	1.762 (3)	C7—C8	1.398 (4)
C1—N1	1.358 (5)	C8—C9	1.378 (4)
C1—C6	1.396 (5)	C8—H8A	0.9300
C1—C2	1.397 (5)	C9—C10	1.386 (4)
N1—H1B	0.8600	C10—C11	1.375 (5)
N1—H1C	0.8600	C10—H10A	0.9300
N2—C7	1.374 (4)	C11—C12	1.368 (5)
N2—H2B	0.8600	C11—H11A	0.9300
N2—H2C	0.8600	C12—H12A	0.9300
C2—C3	1.390 (5)	C13—C14	1.415 (6)
C2—H2D	0.9300	C13—H13A	0.9600
C3—C4	1.371 (5)	C13—H13B	0.9600
C3—H3A	0.9300	C13—H13C	0.9600
C4—C5	1.390 (5)	C14—N3	1.111 (6)
C4—H4A	0.9300		
			
O1—S—O2	118.90 (16)	C5—C6—H6A	119.9
O1—S—C5	108.65 (16)	C1—C6—H6A	119.9
O2—S—C5	107.18 (15)	N2—C7—C12	121.7 (3)
O1—S—C9	108.95 (15)	N2—C7—C8	120.1 (3)
O2—S—C9	107.82 (15)	C12—C7—C8	118.2 (3)
C5—S—C9	104.40 (14)	C9—C8—C7	119.8 (3)
N1—C1—C6	121.8 (3)	C9—C8—H8A	120.1
N1—C1—C2	120.6 (4)	C7—C8—H8A	120.1
C6—C1—C2	117.6 (3)	C8—C9—C10	121.6 (3)
C1—N1—H1B	120.0	C8—C9—S	118.1 (2)
C1—N1—H1C	120.0	C10—C9—S	120.3 (3)
H1B—N1—H1C	120.0	C11—C10—C9	118.1 (3)
C7—N2—H2B	120.0	C11—C10—H10A	121.0
C7—N2—H2C	120.0	C9—C10—H10A	121.0
H2B—N2—H2C	120.0	C12—C11—C10	121.3 (3)
C3—C2—C1	121.5 (4)	C12—C11—H11A	119.3
C3—C2—H2D	119.2	C10—C11—H11A	119.3
C1—C2—H2D	119.2	C11—C12—C7	121.0 (3)
C4—C3—C2	120.5 (4)	C11—C12—H12A	119.5
C4—C3—H3A	119.7	C7—C12—H12A	119.5
C2—C3—H3A	119.7	C14—C13—H13A	109.5
C3—C4—C5	118.3 (3)	C14—C13—H13B	109.5
C3—C4—H4A	120.9	H13A—C13—H13B	109.5
C5—C4—H4A	120.9	C14—C13—H13C	109.5
C6—C5—C4	121.9 (3)	H13A—C13—H13C	109.5
C6—C5—S	119.7 (3)	H13B—C13—H13C	109.5
C4—C5—S	118.3 (3)	N3—C14—C13	179.4 (6)
C5—C6—C1	120.2 (3)		
			
N1—C1—C2—C3	−177.5 (4)	N2—C7—C8—C9	177.1 (3)
C6—C1—C2—C3	0.2 (6)	C12—C7—C8—C9	−0.7 (5)
C1—C2—C3—C4	1.2 (6)	C7—C8—C9—C10	0.0 (5)
C2—C3—C4—C5	−1.5 (6)	C7—C8—C9—S	177.0 (2)
C3—C4—C5—C6	0.6 (5)	O1—S—C9—C8	154.6 (2)
C3—C4—C5—S	−177.8 (3)	O2—S—C9—C8	24.3 (3)
O1—S—C5—C6	11.1 (3)	C5—S—C9—C8	−89.5 (3)
O2—S—C5—C6	140.7 (3)	O1—S—C9—C10	−28.3 (3)
C9—S—C5—C6	−105.0 (3)	O2—S—C9—C10	−158.6 (3)
O1—S—C5—C4	−170.5 (3)	C5—S—C9—C10	87.6 (3)
O2—S—C5—C4	−40.9 (3)	C8—C9—C10—C11	1.2 (5)
C9—S—C5—C4	73.3 (3)	S—C9—C10—C11	−175.8 (3)
C4—C5—C6—C1	0.8 (5)	C9—C10—C11—C12	−1.5 (6)
S—C5—C6—C1	179.1 (3)	C10—C11—C12—C7	0.8 (6)
N1—C1—C6—C5	176.6 (4)	N2—C7—C12—C11	−177.4 (3)
C2—C1—C6—C5	−1.2 (5)	C8—C7—C12—C11	0.4 (5)

### Hydrogen-bond geometry (Å, °)

**Table:**

*D*—H···*A*	*D*—H	H···*A*	*D*···*A*	*D*—H···*A*
C6—H6A···O1	0.93	2.53	2.913 (4)	105
C8—H8A···O2	0.93	2.53	2.906 (4)	104
N1—H1B···O1^i^	0.86	2.32	3.147 (5)	161
N2—H2B···O2^ii^	0.86	2.28	3.079 (4)	155

Symmetry codes: (i) *x*+1/2, *y*, −*z*+1/2; (ii) −*x*+1/2, *y*−1/2, *z*.
